# Hypoxic Preconditioning Maintains GLT-1 Against Transient Global Cerebral Ischemia Through Upregulating Cx43 and Inhibiting c-Src

**DOI:** 10.3389/fnmol.2018.00344

**Published:** 2018-10-01

**Authors:** Kongping Li, Huarong Zhou, Lixuan Zhan, Zhe Shi, Weiwen Sun, Dandan Liu, Liu Liu, Donghai Liang, Yafu Tan, Wensheng Xu, En Xu

**Affiliations:** ^1^Institute of Neurosciences and Department of Neurology of the Second Affiliated Hospital of Guangzhou Medical University and Key Laboratory of Neurogenetics and Channelopathies of Guangdong Province and the Ministry of Education of China, Guangzhou, China; ^2^Department of Environmental Health Sciences, Rollins School of Public Health, Emory University, Atlanta, GA, United States; ^3^Department of Neurology, The First Affiliated Hospital of Guangxi Medical University, Nanning, China

**Keywords:** glutamate transporter 1, glutamine synthetase, connexin 43, c-Src, cerebral ischemia, hypoxic preconditioning, neuroprotection

## Abstract

Transient global cerebral ischemia (tGCI) causes excessive release of glutamate from neurons. Astrocytic glutamate transporter-1 (GLT-1) and glutamine synthetase (GS) together play a predominant role in maintaining glutamate at normal extracellular concentrations. Though our previous studies reported the alleviation of tGCI-induced neuronal death by hypoxic preconditioning (HPC) in hippocampal Cornu Ammonis 1 (CA1) of adult rats, the underlying mechanism has not yet been fully elaborated. In this study, we aimed to investigate the roles of GLT-1 and GS in the neuroprotection mediated by HPC against tGCI and to ascertain whether these roles can be regulated by connexin 43 (Cx43) and cellular-Src (c-Src) activity. We found that HPC decreased the level of extracellular glutamate in CA1 after tGCI via maintenance of GLT-1 expression and GS activity. Inhibition of GLT-1 expression with dihydrokainate (DHK) or inhibition of GS activity with methionine sulfoximine (MSO) abolished the neuroprotection induced by HPC. Also, HPC markedly upregulated Cx43 and inhibited p-c-Src expression in CA1 after tGCI, whereas inhibition of Cx43 with Gap26 dramatically reversed this effect. Furthermore, inhibition of p-c-Src with 4-amino-5-(4-chlorophenyl)-7-(t-butyl) pyrazolo (3, 4-d) pyrimidine (PP2) decreased c-Src activity, increased protein levels of GLT-1 and Cx43, enhanced GS activity, and thus reduced extracellular glutamate level in CA1 after tGCI. Collectively, our data demonstrated that reduced extracellular glutamate induced by HPC against tGCI through preventing the reduction of GLT-1 expression and maintaining GS activity in hippocampal CA1, which was mediated by upregulating Cx43 expression and inhibiting c-Src activity.

## Introduction

Global cerebral ischemia (GCI) may be caused by acute heart failure, cardiac arrest and shock etc., and lead to delayed neuronal damage in the hippocampal Cornu Ammonis 1 (CA1) subregion. Transient episodes of nonlethal pretreatments have been proven to confer profound protection on the neurons in response to a prolonged lethal episode of ischemia-reperfusion (I/R), a phenomenon that has been termed “preconditioning or ischemic tolerance.” We previously reported that hypoxic preconditioning (HPC) with 8% oxygen for 30–120 min applied 1–4 days before ischemia reduced cell death in CA1 after 10 min of transient GCI (tGCI). The maximum protection was observed with 30 min of hypoxia and 1-day interval between hypoxia and tGCI (Zhan et al., 2010). Even so, the underlying molecular mechanisms by which HPC intervenes in tGCI-induced cell death are not fully understood.

Glutamate, physiologically an important excitatory neurotransmitter, plays an essential role in the body, and any excess release of glutamate can be dangerous. As a proven fact, the astrocytic glutamate transporters-1 (GLT-1) are deemed critical for powerful control on maintaining glutamate homeostasis by removing excessive synaptic glutamate to prevent excitotoxicity (Kim et al., 2011; Kostandy, 2012). Accumulating evidences indicate that focal or global cerebral ischemia would decrease GLT-1 expression in glial cells, which might impair normal clearance of glutamate and contribute to neuronal damage (Yeh et al., 2005; Ketheeswaranathan et al., 2011). No less importantly, the up-regulation of GLT-1 by genetic or pharmacological strategy dramatically decreases extracellular glutamate and protects neurons against glutamate excitotoxicity (Verma et al., 2010; Harvey et al., 2011). Under ischemic conditions, the released glutamate from neurons and astrocytes shall be mainly taken up into astrocytes by GLT-1 and converted to glutamine by glutamine synthetase (GS), a key enzyme for the regulation of the extracellular glutamate in the glutamate-glutamine cycle localized primarily in astrocytes. This disorder of glutamate-glutamine cycle related to a metabolic trafficking mechanism between glia and neurons plays an important role in neuronal death after cerebral ischemia (Wang et al., 2013; Momosaki et al., 2015). The loss of GS would increase the level of brain glutamate (Wang et al., 2013). What still remains unclear is whether the expression of GLT-1 and/or activity of GS is related to HPC-induced neuroprotection against tGCI.

Based on the crucial role of GLT-1 in maintaining brain function, numerous studies have explored the mechanisms regulating the expression of GLT-1 (Figiel et al., 2007; Unger et al., 2012). It is known that connexins are transmembrane proteins widely distributed in the body and are crucially important for heart and brain (Schulz et al., 2015). Among these proteins, connexins 43 (Cx43) plays a key role in building a block of gap junction (GJ) hemichannels in astrocytes (Li et al., 2015; Tabernero et al., 2016). Decreased expression of Cx43 would lead to the downregulation of GLT-1 and subsequently enhance synaptic glutamatergic neurotransmission (Figiel et al., 2007; Morioka et al., 2015). On the contrary, other reports showed that the decline or loss of Cx43 was associated with the increased expression of GLT-1 in the hippocampus of rats post traumatic brain injury or in cerebral cortex of mice with Cx43 knockout (Unger et al., 2012; Sun et al., 2015). It is still unclear how Cx43 regulates expression of GLT-1 and further affects cell survival in the HPC mediated neuroprotection against tGCI.

The intracellular carboxyl tail of Cx43 interacts with a number of scaffolding protein, thereby regulating cell functions. One of these interacting proteins is cellular-Src (c-Src; Giepmans et al., 2001; González-Sánchez et al., 2016), a non-receptor proto-oncogene tyrosine-protein kinase that participates in different biological events, including proliferation, differentiation, survival and migration (Thomas and Brugge, 1997). Evidences are provided that rapid activation of c-Src played a central role in executing glutamate-induced neurodegeneration (Khanna et al., 2007) and knockdown of c-Src protected cells against glutamate-induced loss of viability (Khanna et al., 2005). Therefore, c-Src is associated with cell death pathways in neurons subjected to glutamate excitotoxic insults. However, the roles of Cx43 and c-Src on the regulation of GLT-1 and GS in the HPC mediated neuroprotection against tGCI remain to be elucidated.

To fill the knowledge gaps, we conducted the present study to test the hypothesis that decreasing concentration of extracellular glutamate through preventing reduction of GLT-1 expression and maintaining GS activity, which is mediated by Cx43 and c-Src, contributed to the HPC mediated neuroprotection against tGCI in adult rats.

## Materials and Methods

### Animals

Animal experiments were performed on adult male Wistar rats aging 20–25 weeks, weighing 220–280 g (Southern Medical University, Guangdong, China). Rats were treated in accordance with the Guide for the Care and Use of Laboratory Animals (NIH Publication No. 80–23, Revised 1996). Experimental protocols were approved and monitored by the Animals Care and Use committee of Guangzhou Medical University. All efforts were made to reduce the number of animals used and minimize animal suffering.

### Transient Global Ischemia and Hypoxic Preconditioning

According to the four-vessel occlusion method (Pulsinelli and Brierley, 1979), tGCI was carried out. All procedures were performed under aseptic conditions. Briefly, the animals were anesthetized with chloral hydrate (350 mg/kg, intraperitoneally). Vertebral arteries were electrocauterized, and common carotid arteries were isolated. A teflon/silastic occluding device was placed loosely around each carotid artery without interrupting carotid blood flow. Forebrain ischemia was induced 24 h after surgery in the awake rats via 10-min occlusion of bilateral common carotid arteries. After occlusion, rats that lost their righting reflex within 1 min and whose pupils dilated were selected for experiments. Rectal temperature was maintained at 37–38°C throughout the procedure. Sham-operated (Sham) rats received the same surgical procedure except the occlusion of arteries. Rats that convulsed during ischemia or post-ischemia were excluded from this study.

Twenty-four hours before ischemia, rats were placed in a hypoxic chamber through which air containing 8% O_2_ and 92% N_2_ flowed continuously at the temperature of 23–25°C and preconditioned for 30 min (Zhan et al., 2010).

### Brain Microdialysis

At 5 days before the experiment, two guide cannulas were implanted stereotaxically under anesthesia with chloral hydrate (350 mg/kg, intraperitoneally). One guide cannula (CXG-2, Eicom, Japan) was implanted into the right dorsal hippocampal CA1 region (3.5 mm posterior to Bregma, 2.3 mm lateral to Bregma, 2.5 mm below the dura) for microdialysis. The other was implanted into the left lateral ventricle for drugs administration through a burr hole opened on the parietal skull at 1.5 mm lateral, 0.8 mm posterior and 4.0 mm dorsal with respect to the Bregma. The guide cannulas were secured to the skull with screws and dental cement. Rats were allowed to recover from surgery for 5 days before treatment.

Artificial cerebrospinal fluid (aCSF, containing NaCl 149 mmol/L, KCl 3.9 mmol/L, MgCl_2_ 20.8 mmol/L, CaCl_2_ 21.2 mmol/L) was perfused through the dialysis probes (Cx-I-2-03, Eicom, Japan). The flow rate was fixed at 2.0 μL/min throughout the procedure of microdialysis. After 1-h stabilization, 10 min before the corresponding time point (0, 4, 24 and 48 h), microdialysate samples were collected with refrigerated fraction collector (EFC-96, Eicom, Japan) for 20 min. Only samples collected from rats in which the probe was verified to be located in the CA1 were used for analysis.

### Determination of Extracellular Glutamate

According to the previously described protocol (Spink et al., 1986), extracellular glutamate in the microdialysate was analyzed by O-phthalaldehyde (OPA) precolumn derivatization using high-performance liquid chromatography (HPLC) system (HP 1050, USA) with fluorescence detection in National Analytical Center of China (NACC), Guangzhou. Briefly, One microliters of microdialysate, 2.0 μL borate buffer solution (pH 10.05) and 2.0 μL OPA solution were mixed and reacted for 1 min at room temperature, and then 1.0 μL 9-fluolenylmeghyl chloroformate (FMOC) was added into mixture and reacted for 1 min at room temperature. After reacting, a 6-μL aliquot of the reaction mixture was injected into the HPLC system with an autosampler. The separation was performed on a Hypersil ODS chromatographic column (5 μm particle size, 4.0 × 125 mm) with the mobile phases of 10 mM Na_2_HPO_4_-NaH_2_PO_4_ buffer (PB, pH 7.2, including 0.5% (ϕ) tetrahydrofuran (THF), (phase A) and PB-methanol-Methyl Cyanide (volume ratio, 50:35:15; phase B). The analysis was performed with linear gradient from A:B (100:0) to 100% B within 0–25 min; then eluted with 100% B for 5 min to elute other components. The flow rate was 1.0 mL/min. The excitation and emission wave lengths were 340 and 450 nm, respectively. Chromatographic peaks of glutamate were identified in accordance with the standards containing known amounts of glutamate. Sets of standards were run before each sample analysis.

### Immunohistochemistry

The rats were sacrificed at 0, 4, 24, 48 and 168 h after reperfusion with or without hypoxia (*n* = 6 in each group), respectively. Single-label immunohistochemistry was performed as described previously (Zhan et al., 2010). Briefly, sections were first treated with 3% H_2_O_2_ for 30 min, followed by 5% normal serum for 1 h, and they were then incubated overnight at 4°C with primary antibodies including a mouse monoclonal antibody against NeuN (1:8,000; Millipore, Cat# MAB377, RRID: AB_2298772), a Guinea Pig polyclonal antibody against GLT-1 (1:2,000; Millipore, Cat# AB1783, RRID: AB_90949), a mouse monoclonal antibody against GS (1:2,000; Millipore, Cat# MAB302, RRID: AB_2110656), a mouse monoclonal antibody against Cx43 (1:100; Millipore, Cat# MAB3067, RRID: AB_94663), a rabbit monoclonal antibody against c-Src (1:4,000; Cell Signaling Technology, Cat# 2109, RRID: AB_2106059) and a rabbit monoclonal antibody against c-Src phosphorylated at tyrosine 416 (p-c-Src; 1:1,000; Cell Signaling Technology, Cat# 6943, RRID: AB_10013641). All antibodies except for GLT-1 which was from Guinea Pig were prepared from rabbits or mouse. The slides were washed with 0.01 M phosphate buffer saline (PBS, pH = 7.4) for three times, and then were incubated with biotinylated secondary immunoglobulin G antibody for 2 h at room temperature. After being washed with PBS, the sections were incubated with the avidin-biotin-peroxidase complex for 30 min at room temperature. The peroxidase reaction was visualized with 0.05% diaminobenzidine and 0.01% hydrogen peroxide. Immunopositive cells in which the reaction product was present within a clear and regular-shaped cytoplasmic or nuclear border were quantified under a light microscope with magnification (×660). The total numbers of GS and p-c-Src immunopositive cells in the CA1 pyramidal layer were quantitatively analyzed within three non-repeated rectangular areas of 0.037 mm^2^, respectively. The average intensity of GLT-1 and Cx43 in which the reaction product was in the cell processes in CA1 was determined using the Image-Pro Plus software for Windows, version 6.0 (Media Cybernetics, Inc., Warrendale, PA, USA). Four non-repeated random fields (141.15 μm^2^ per field) under a light microscope with magnification (×200) in the pyramidal layer, stratum radiatum, and stratum lacunosum-moleculare of each rat were assessed in four coronal tissue sections. Measures of the mean optical densities in GLT-1 and Cx43 immunopositive staining were averaged across tissue sections to provide a single mean value for each rat. These mean values were used for statistical analysis.

Double-fluorescent immunohistochemistry was performed as described previously (Zhan et al., 2010). Neuronal nuclei (NeuN), glial fibrillary acidic protein (GFAP) and ionized calcium binding adaptor molecule-1 (Iba-1) were used to identify NeuN, astrocytes and microglia, respectively. Antibodies used in these studies included a Guinea Pig polyclonal antibody against GLT-1 (1:50; Millipore, Cat# AB1783, RRID: AB_90949), a mouse monoclonal antibody against Cx43 (1:50; Abcam, Cat# ab79010, RRID: AB_1603627), a rabbit monoclonal antibody against c-Src phosphorylated at tyrosine 416 (p-c-Src; 1:50; Cell Signaling Technology, Cat# 6943, RRID: AB_10013641), a rabbit polyclonal antibody against NeuN (1:1,000; Millipore, Cat# ABN78, RRID: AB_10807945), a mouse monoclonal antibody against NeuN (1:1,000; Millipore, Cat# MAB377, RRID: AB_2298772), a rabbit polyclonal antibody against GFAP (1:3,000; Millipore, Cat# AB5804, RRID: AB_11212369), a mouse monoclonal antibody against GFAP (1:3,000; Millipore, Cat# MAB360, RRID: AB_2109815), a mouse monoclonal antibody against Iba-1 (1:100, Wako, Cat# 016–26721), a rabbit polyclonal antibody against Iba-1 (1:100, Abcam, Cat# ab108539, RRID: AB_10862652), Cy3-conjugated goat anti-mouse IgG antibody (1:100; Millipore, Cat# AP124C, RRID: AB_11213281), FITC-conjugated goat anti-rabbit polyclonal antibody (1:100; Millipore, Cat# AP307F, RRID: AB_92652), Cy3-conjugated goat anti-rabbit IgG antibody (1:100; Millipore, Cat# AP132C, RRID: AB_92489), FITC-conjugated goat anti-mouse polyclonal antibody (1:100; Millipore, Cat# AP308F, RRID: AB_92634) and FITC-conjugated goat anti-Guinea Pig IgG polyclonal antibody (1:100; Millipore, Cat# AP108F, RRID: AB_11210667). And then, they were washed with PBS and mounted with mounting medium containing 4’,6-diamidino-2-phenylindole (DAPI). Slides were analyzed with a confocal laser microscope (XP8, Leica Microsystems, Wetzlar, Hessen, Germany). The total number of GLT-1, Cx43, p-c-Src positive cells and GLT-1, Cx43, p-c-Src positive neurons or astrocytes or microglia was also counted within three non-repeated rectangular areas of 0.037 mm^2^ in the CA1 regions, respectively.

Triple-fluorescent immunohistochemistry was performed as previously described (Zuo et al., 2018). Sections were preincubated with 5% normal serum (containing 0.2% Triton X-100) for 1 h at room temperature, and then incubated overnight at 4°C with mixtures of primary antibodies: goat polyclonal antibody against Cx43 (1:100; Abcam, Cat# ab219493), mouse monoclonal antibody against NeuN (1:1,000; Millipore, Cat# MAB377, RRID: AB_2298772), rabbit polyclonal antibody against Iba-1 (1:100, Abcam, Cat# ab108539, RRID: AB_10862652). After rinsing in 0.01 M PBS, the sections were incubated for 2 h at room temperature with the following secondary antibodies: Cy3-conjugated goat anti-rabbit IgG antibody (1:100; Millipore, Cat# AP132C, RRID: AB_92489), FITC-conjugated goat anti-mouse polyclonal antibody (1:100; Millipore, Cat# AP308F, RRID: AB_92634), and donkey anti-goat IgG H&L (Alexa Fluor^®^ 405) antibody (1:100, Abcam, Cat# ab175664, RRID: AB_2313502). Slides were analyzed with a confocal laser microscope (XP8, Leica Microsystems, Wetzlar, Hessen, Germany).

### Western Blot

Rats were sacrificed at 0, 24, and 48 h after reperfusion with or without hypoxia (*n* = 3 in each group), respectively. The CA1 subregion protein extraction was performed as described previously (Yano et al., 2001). To determine protein concentration, bicinchoninic acid (BCA) method was recommended by the manufacturer (Beyotime, Jiangsu, China). Fifty micrograms proteins of each sample were separated by sodium dodecyl sulfatepolyacrylamide gel electrophoresis (SDS-PAGE) using 10% acrylamide gels, and then transferred to polyvinylidene fluoride (PVDF) membranes (Millipore, MA, USA). Western blotting analyses were performed as described previously (Endo et al., 2006). Primary antibodies included a rabbit polyclonal antibody against GLT-1 (1:1,000; Cat# ABN102, Millipore, MA, USA), a mouse monoclonal antibody against GS (1:20,000; Millipore, Cat# MAB302, RRID: AB_2110656), a mouse monoclonal antibody against Cx43 (1:4,000; Millipore, Cat# MAB3067, RRID: AB_94663), a rabbit monoclonal antibody against c-Src (1:10,000; Cell Signaling Technology, Cat# 2109, RRID: AB_2106059) and a rabbit monoclonal antibody against c-Src phosphorylated at tyrosine 416 (p-c-Src; 1:2,000; Cell Signaling Technology, Cat# 6943, RRID: AB_10013641) and a mouse monoclonal antibody against GADPH (Proteintech Group, Cat# 60004-I-Ig, RRID: AB_2107436). Densitometry analysis for the quantification of the bands was performed using the Quantity One 1-D Analysis Software (Quantity One, Bio-Rad Laboratories, Inc., Hercules, CA, USA). Relative optical densities of protein bands were calibrated with GAPDH and normalized to those in Sham rats.

### Histology

Rats were perfused intracardially with 0.9% normal saline, followed by 4% paraformaldehyde in PBS under anesthesia at 168 h after ischemia. The brains were removed quickly and further fixed with the same solution at 4°C overnight. Postfixed brains were immersed in 10%, 20% and 30% sucrose in the same fixative for cytoprotection and were cut into 30-μm thick slices using a cryotome (Thermo, Runcorn, Cheshire, UK). The dorsal hippocampus sections (between AP 4.8 and 5.8 mm, interaural or AP-3.3–3.4 mm, Bregma) were selected. As studied previously (Zhan et al., 2012), Nissl and Fluoro-Jade B (FJ-B) staining were performed to determine the hippocampal cell damage. Nissl stained sections were observed under a light microscope with magnification (×660). Nissl staining was performed with 0.1% cresyl violet (Sigma, St. Louis, MO, USA). Survived cells displayed well-stained Nissl bodies, whereas damaged cells were either swollen with loss of stainable Nissl material or necrotic with deeply staining dendrites fragmented. FJ-B stained images for detecting degenerating cells were analyzed with a fluorescence microscope (Leica Microsystems, Wetzlar, Hessen, Germany). Cell counts were conducted as described previously (Wang et al., 2011). Cells in the CA1 pyramidal layer were quantitatively analyzed within three non-repeated rectangular areas of 0.037 mm^2^. Data were quantified bilaterally in sections from each brain and assessed double-blindedly. Besides, four sections for each rat were evaluated.

### Assay of Glutamine Synthetase Activity

The GS activity in CA1 was measured by spectrophotometric method (Beijing Solarbio Science & Technology Co., Ltd, China). Briefly, the tissues from CA1 of rats subjected to tGCI with or without HPC after 0, 4, 24 and 48 h (*n* = 6 in each group) of reperfusion were homogenized in extracting solution. The homogenates were centrifuged at 8,000 *g* for 10 min at 4°C. The supernatants were used for further processing. The samples (0.07 mL) were incubated with 0.23 mL of the reaction mixture consisting of 0.16 mL of reagent I and 0.07 mL of reagent III for 30 min at 37°C. The chromogenic reaction was worked by adding 0.1 mL of reagent IV. After mixing in platform vibrator for 10 min on ice, the mixture was centrifuged at 8,000 *g* for 10 min at room temperature. The absorbance of the supernatant was measured at 540 nm and compared to the corresponding reagent blanks. The unit of GS activity was expressed as a 0.005 change of absorption value under 540 nm per min per mL per g of protein. For the final calculation, GS activity of each group was expressed as a percentage of the Sham control.

### Drug Injection

For pharmacologic interventions, the specific GLT-1 inhibitor dihydrokainic acid (DHK, 2 nmol and 4 nmol, respectively; Tocris Bioscience, USA and Canada, 200 μmol/L in 0.01 M PBS; John et al., 2015; Zhang M. et al., 2007), Gap26 (a connexin mimetic peptide, 10 μL and 20 μL, respectively; BIOPIKE, 300 μmol/L in 0.01 M PBS; Sun et al., 2012), or the vehicle (25% dimethylsulfoxide (DMSO) in PBS) was injected intracerebroventricularly at 30 min before HPC (ICV, Bregma:1.5 mm lateral, 0.8 mm posterior, 4.0 mm deep). L-methionine-DL-sulfoximine (methionine sulfoximine (MSO), GS inhibitor, Sigma Aldrich, St. Louis, MO, USA; Trabelsi et al., 2017) was dissolved in 0.9% normal saline and administered intraperitoneally in a dose of 170 mg/kg at 2 h before HPC. Sham animals were injected only with normal saline. A selective Src family kinase inhibitor 4-amino-5-(4-chlorophenyl)-7-(t-butyl) pyrazolo (3, 4-d) pyrimidine (PP2, 10 μL and 20 μL, repectively; EMD Chemicals, Inc. San Diego, USA and Canada, 3 mg/L in 100% DMSO; Lennmyr et al., 2004; Hou et al., 2007), or the vehicle (25% DMSO in PBS) was administered at 24 h before tGCI. To evaluate the cytotoxicity of abovementioned inhibitors to CA1, DHK, Gap26 and PP2 were injected intracerebroventricularly and MSO injected intraperitoneally to Sham animals.

### Statistical Analysis

Statistical analyses were performed with the Statistical Package for Social Sciences Software for Windows, version 13.0 (SPSS, Inc., Chicago, IL, USA). Results were presented as the mean ± standard deviation (SD). Statistical significance was determined by one-way ANOVA analysis followed by a Bonferroni or Tamhane’s T2 *post hoc* test. Statistical significance was considered to be *p* < 0.05.

## Results

In the present study, 476 rats were used for the experiments, six of which in tGCI group and five in HPC group died during the process of ischemia; four in tGCI group and two in HPC group died during reperfusion; four died after intraperitoneal injection; four died after intracerebroventricular injection and six died after microdialysis.

### Hypoxic Preconditioning Decreases the Accumulation of Extracellular Glutamate by Maintaining GLT-1 Expression After tGCI

**Figure 1:**
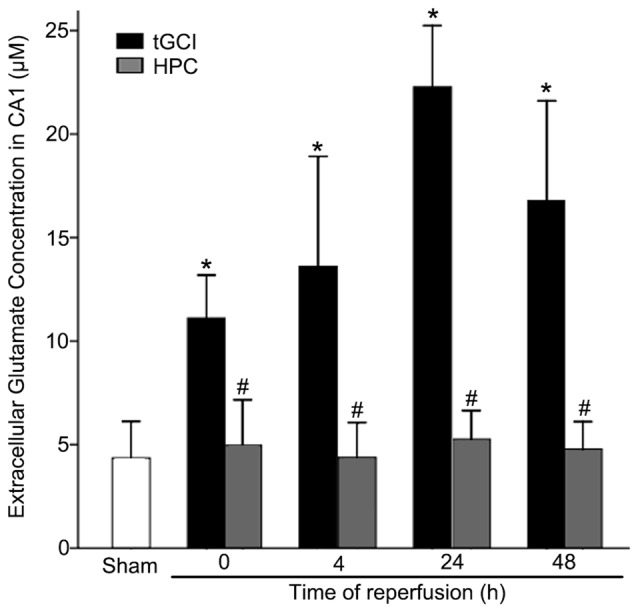
HPC prevents the extracellular glutamate accumulation in hippocampal Cornu Ammonis 1 (CA1) region after tGCI. Glutamate content of the dialysates was analyzed using high-performance liquid chromatography (HPLC) system with electrochemical detection. The histogram presents the quantitative analyses of extracellular glutamate content in CA1. Each bar represents the mean ± S.D. *p < 0.05 vs. Sham group and ^#^*p* < 0.05 vs. tGCI group at the same time point (*n* = 4 in each group). μM, μmol/L; Sham, sham-operated; tGCI, transient global cerebral ischemia; HPC, hypoxic preconditioning.

The content of extracellular glutamate in hippocampal CA1 of rats was measured with microdialysis. As showed in Figure 1, compared with Sham group, the level of extracellular glutamate in CA1 of tGCI increased as early as 0 h after reperfusion, peaked at 24 h, and maintained at a high level for at least 48 h. On the contrary, HPC prevented the accumulation of extracellular glutamate after tGCI.

**Figure 2:**
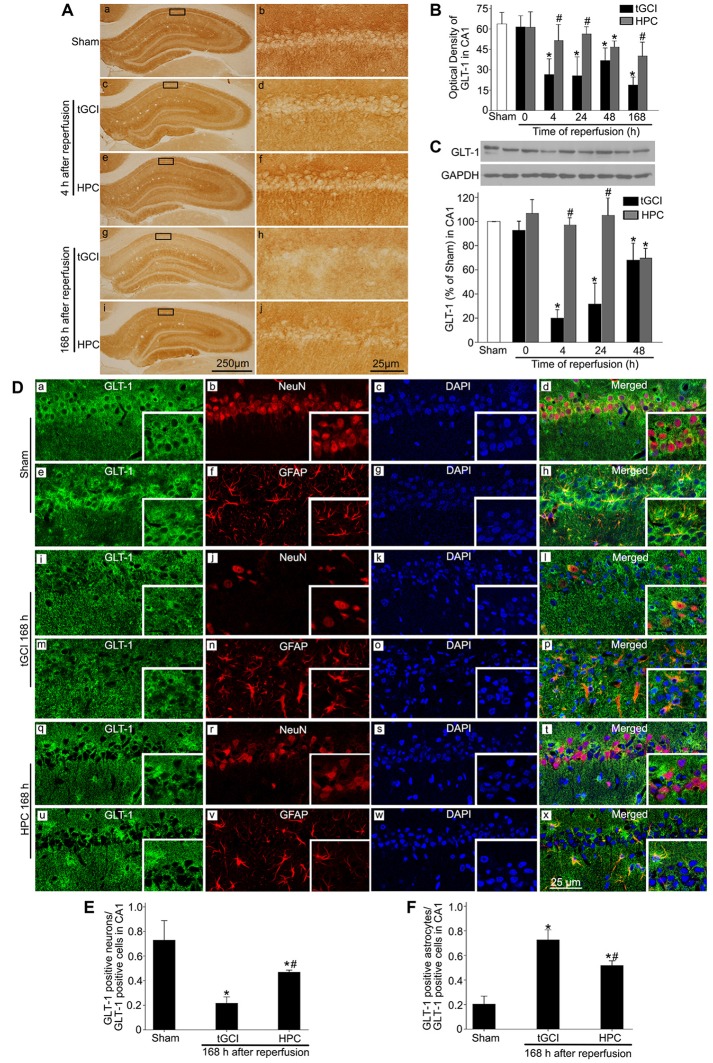
HPC regulates GLT-1 expression in CA1 after tGCI. **(A)** Immunohistochemistry for GLT-1 in hippocampus after tGCI with or without HPC. Representative images show Sham group **(a,b)**, 4 h after reperfusion of tGCI group **(c,d)** and HPC group **(e,f)**, and 168 h after reperfusion of tGCI group **(g,h)** and HPC group **(i,j)**, respectively. Scale bar: **(a,c,e,g,i)**: 250 μm; **(b,d,f,h,j)**: 25 μm. **(B)** Quantitative analysis of optical density of GLT-1 immunopositive cells in CA1 (*n* = 6 in each group). **(C)** Western blot analysis of GLT-1 in CA1. The histogram presents the quantitative analyses of GLT-1 levels (*n* = 3 in each group). Data are expressed as percentage of value of Sham animals. Each bar represents the mean ± S.D. *p < 0.05 vs. the Sham animals and ^#^*p* < 0.05 vs. the tGCI group at the same time point. **(D)** Cellular localization of GLT-1 in CA1. **(a–h)** Representative images of triple fluorescent staining of GLT-1 (green), NeuN (red) and DAPI (blue); GLT-1 (green), GFAP (red) and DAPI (blue) in CA1 of Sham group. The overlapped images show that GLT-1 immunopositive cells mainly surrounded NeuN, whereas partially colocalized with GFAP. **(i–p)** Representative images of GLT-1 (green), NeuN (red) and DAPI (blue); GLT-1 (green), GFAP (red) and DAPI (blue) in CA1 of tGCI group at 168 h after reperfusion. GLT-1 located mainly in GFAP-positive cells. **(q–x)** Representative images of GLT-1 (green), NeuN (red) and DAPI (blue); GLT-1 (green), GFAP (red) and DAPI (blue) in CA1 of HPC group at 168 h after reperfusion. GLT-1 located mainly in NeuN- and GFAP-positive cells at 168 h after HPC. Scale bar: 25 μm. **(E,F)** Quantitative analysis of GLT-1-positive neurons and GLT-1-positive astrocytes in CA1. Data are expressed as percentage of the number of GLT-1-positive cells. Each bar represents the mean ± S.D. *p < 0.05 vs. Sham group and ^#^*p* < 0.05 vs. tGCI group (*n* = 4 in each group). Sham, sham-operated; tGCI, transient global cerebral ischemia; HPC, hypoxic preconditioning; DAPI, 4’,6-diamidino-2-phenylindole; GLT-1, glutamate transporter 1; NeuN, neuronal nuclei; GFAP, glial fibrillary acidic protein.

To ascertain the role of GLT-1 in regulating extracellular glutamate in HPC rats, the expression of GLT-1 in CA1 was measured with immunohistochemical assay. It was demonstrated that there were more neuron-like cells tightly surrounded by GLT-1 immuno-particles in the HPC groups, resembling “shaped grid” in the pyramidal layer (Figures 2Aa–j). Compared to Sham group, GLT-1 immunoreactivity sharply decreased at all time points in tGCI groups, while HPC thoroughly prevented this reduction induced by tGCI (Figure 2B). Also, the changes in GLT-1 level measured by western blot were well correlated with the immunohistochemical assay (Figure 2C).

Triple-fluorescent immunohistochemical studies showed that the cells surrounded by GLT-1 positive labellings were primarily NeuN-positive (Figures 2Da–d) and that a few GLT-1 positive cells were co-localized with GFAP (Figures 2De–h) in sham-operated group, indicating that GLT-1 was predominantly localized in the cytoplasms and dendrites of neurons and slightly in the processes of astrocytes. However, at 168 h after reperfusion in tGCI, most of GLT-1 positive cells were co-localized with GFAP (Figures 2Di–l), suggesting that GLT-1 was predominantly localized in astrocytes in tGCI rats. In tGCI rats, the activated astrocytes displayed an appearance with hypertrophic cell bodies, thick and prolonged processes (Figures 2Dm–p). Alternatively, some GLT-1 positive labellings around NeuN-positive cells were observed in HPC rats (Figures 2Dq–t). In addition, most astrocytes exhibited quiescent phenotype with a stellate, process-bearing shape at 168 h after reperfusion in HPC rats (Figures 2Du–x). As shown in Figures 2E,F, the quantitative analysis showed that HPC increased the number of GLT-1 positive neurons and decreased the GLT-1 positive astrocytes at 168 h after tGCI.

**Figure 3:**
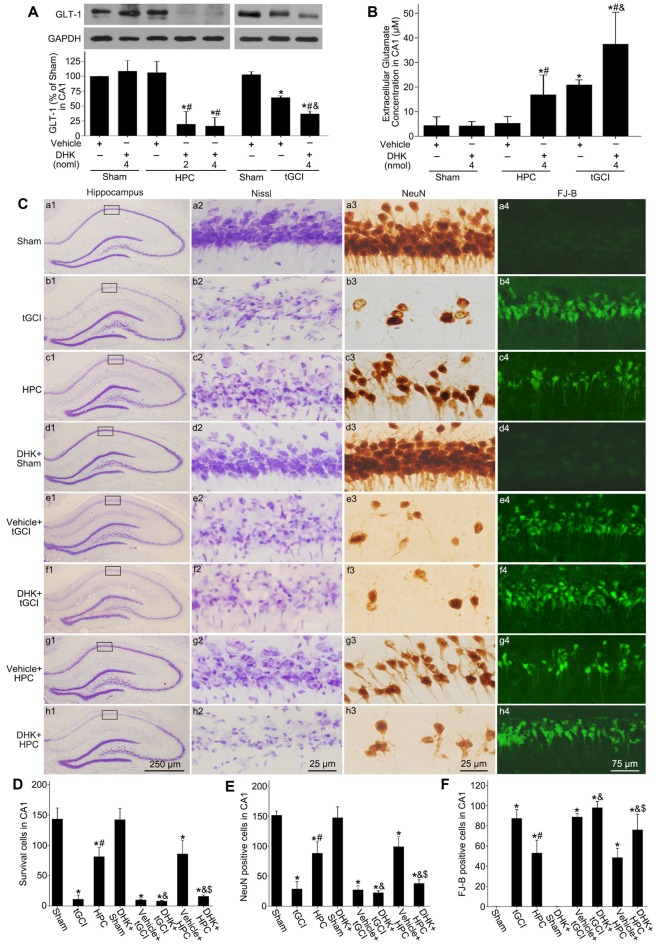
GLT-1 inhibition with DHK aggravates neuronal damage and increases the extracellular glutamate level in CA1 of HPC rats.** (A)** Effect of DHK pretreatment (2 nmol and 4 nmol, respectively) on the expression of GLT-1 in CA1 at 4 h after reperfusion of tGCI with or without HPC. **(B)** Effect of DHK pretreatment (4 nmol) on extracellular glutamate level in CA1 at 24 h of reperfusion of tGCI with or without HPC. Each bar represents the mean ± S.D. *p < 0.05 vs. Sham group pretreated with vehicle, ^#^*p* < 0.05 vs. the same group pretreated with vehicle, and ^&^*p* < 0.05 vs. HPC group pretreated with DHK (*n* = 3 in each group). **(C)** Nissl staining, NeuN immunostaining and FJ-B staining in hippocampus of HPC and tGCI rats with or without DHK treatment at 168 h after reperfusion. Boxes indicated that the magnified regions displayed in the right panel. Scale bar: **(a1,b1,c1,d1,e1,f1,g1,h1)** 250 μm; **(a2,a3,b2,b3,c2,c3,d2,d3,e2,e3,f2, f3,g2,g3,h2,h3)** 25 μm; **(a4,b4,c4,d4,e4,f4,g4,h4)** 75 μm. Quantitative analyses of survival cells **(D)**, NeuN-positive cells **(E)** and FJ-B-positive cells **(F)** in CA1 region. Each bar represents the mean ± S.D. *p < 0.05 vs. Sham animals, ^#^*p* < 0.05 vs. tGCI group, ^&^*p* < 0.05 vs. the same group pretreated with vehicle, and ^$^*p* < 0.05 vs. tGCI group pretreated with DHK (*n* = 6 in each group). Sham, sham-operated; HPC, hypoxic preconditioning; tGCI, transient global cerebral ischemia; GLT-1, glutamate transporter 1; DHK, dihydrokainate; Nissl, cresyl violet; NeuN, neuronal nuclei; FJ-B, Fluoro-Jade B.

To further confirm the reduction of extracellular glutamate induced by HPC after tGCI was involved in the neuroprotection by maintaining GLT-1 expression, inhibitory experiments with DHK were conducted. Western blot analysis showed that GLT-1 expression in CA1 decreased at 4 h after reperfusion with DHK pretreatment in HPC and tGCI groups when compared to vehicle administration. No significant differences in the inhibitory effect of DHK on the GLT-1 expression were noticed between two dosage (2 nmol and 4 nmol) groups in HPC rats, and between Sham groups with or without DHK (Figure 3A). Moreover, the pretreatment with DHK led to a significant increase in extracellular glutamate level in HPC and tGCI rats, and the latter was more noticeable (Figure 3B). Delayed neuronal death was evaluated on 168 h after ischemia with or without DHK. No significant neuronal loss was detected in CA1 region of rats subjected to sham operation (Figures 3Ca1–4), while obvious neuronal damage and loss of pyramidal neurons were observed in CA1 of the tGCI group (Figures 3Cb1–4). When rats were pretreated with hypoxia before tGCI, the delayed neuronal death induced by tGCI was prevented (Figures 3Cc1–4). DHK in a dose of 4 nmol had no effect on the pyramidal neurons of hippocampus in Sham rats (Figures 3Cd1–4). In addition, compared with the vehicle groups before tGCI or HPC (Figures 3Ce1–4,g1–4), serious delayed neuronal death was observed in rats with DHK pretreatment (Figures 3Cf1–4,h1–4), with critical decreased surviving cells and NeuN-positive cells and increased FJ-B-positive cells (Figures 3D–F). These results revealed that the inhibition of GLT-1 expression in CA1 could increase extracellular glutamate and block the HPC mediated neuroprotection against tGCI.

### Hypoxic Preconditioning Decreases the Accumulation of Extracellular Glutamate by Enhancing GS Activity After tGCI

**Figure 4:**
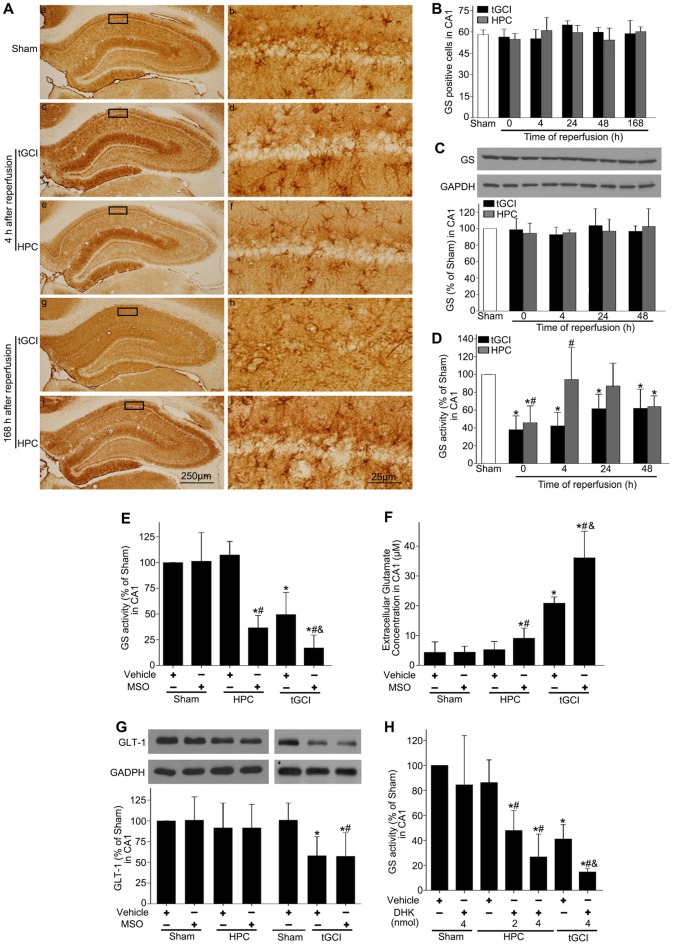
HPC enhances GS activity and reduces the extracellular glutamate level in CA1 after tGCI. **(A)** Immunohistochemistry for GS in hippocampus after tGCI with or without HPC. Representative images show GS-immunopositive cells in Sham group **(a,b)** 4 h after reperfusion of tGCI group **(c,d)** and HPC group **(e,f)** and 168 h after reperfusion of tGCI group **(g,h)** and HPC group **(i,j)** respectively. Scale bar: **(a,c,e,g,i)** 250 μm; **(b,d,f,h,j)** 25 μm. **(B)** Quantitative analysis of GS immunopositive cell in CA1 (*n* = 6 in each group). **(C)** Western blot analysis of GS protein level in CA1 of tGCI and HPC rats. The histogram presents the quantitative analyses of GS level. Data are expressed as percentage of value of Sham animals (*n* = 3 in each group). **(D)** Quantitative analyses of GS activity in CA1 of tGCI and HPC rats. Data are expressed as percentage of value of Sham animals (*n* = 6 in each group). Each bar represents the mean ± S.D. *p < 0.05 vs. the Sham animals and ^#^*p* < 0.05 vs. tGCI group at the same time point. Effect of MSO pretreatment on GS activity **(E)** and expression of GLT-1 **(G)** in CA1 at 4 h of reperfusion of tGCI with or without HPC. **(F)** Effect of MSO pretreatment on extracellular glutamate in CA1 at 24 h of reperfusion of tGCI with or without HPC (*n* = 3 in each group). **(H)** Effect of DHK pretreatment on GS activity in CA1 at 4 h of reperfusion of tGCI with or without HPC (*n* = 4 in each group). Each bar represents the mean ± S.D. *p < 0.05 vs. sham group pretreated with vehicle, ^#^*p* < 0.05 vs. the same group pretreated with vehicle and ^&^*p* < 0.05 vs. HPC group pretreated with MSO **(E–G)** or DHK (**H**; *n* = 3 in each group). Sham, sham-operated; tGCI, transient global cerebral ischemia; HPC, hypoxic preconditioning; GS, glutamine synthetase; MSO, methionine sulfoximine; GLT-1, glutamate transporter 1; DHK, dihydrokainate.

Through immunohistochemistry, we found that GS immunopositive cells displayed an irregular appearance with polymorphic somata and processes (Figures 4Aa–j). Quantitative analysis showed no significant difference in the number of GS-immunopositive cells in CA1 from ischemic or HPC brains up to 168 h of reperfusion (Figure 4B). Similarly, no significant difference was observed in GS protein level among sham-operation, tGCI and HPC groups at different time points (Figure 4C). Further, we observed a significant decrease in GS activity after tGCI. However, the decrease in GS activity at 0 h and 4 h after reperfusion of tGCI was reversed by HPC (Figure 4D).

To determine the impact of GS activity on the content of extracellular glutamate after tGCI with or without hypoxia, MSO was preadministered intraperitoneally. As shown in Figure 4E, MSO led to an almost 3-fold decrease of GS activity in CA1, while Figure 4F exhibited a significant increase in the extracellular glutamate level in HPC and tGCI rats, and the latter was much more obvious.

To assess the connection between GLT-1 upregulation and enhanced GS activity in neuroprotection mediated by HPC, rats were pretreated with either MSO or DHK. As shown in Figure 4G, MSO had no effects on the expression of GLT-1 compared with vehicle in Sham, tGCI or HPC groups, while DHK decreased the activity of GS in the rats of both tGCI and HPC groups in comparison to vehicle, and this declining trend was prominent in tGCI rats (Figure 4H).

### Hypoxic Preconditioning Maintains Cx43 Expression and Inactivates c-Src After tGCI

**Figure 5:**
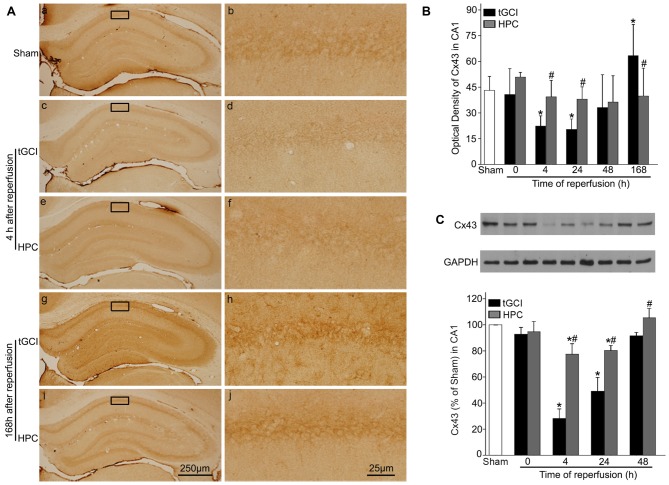
HPC increases the Cx43 expression in CA1 after tGCI. **(A)** Immunohistochemistry for Cx43 in hippocampus after tGCI with or without HPC. Representative images show Cx43-immunopositive cells in Sham group **(a,b)**, 4 h after reperfusion of tGCI group **(c,d)** and HPC group **(e,f)**, and 168 h after reperfusion of tGCI group **(g,h)** and HPC group **(i,j)**, respectively. Scale bar: **(a,c,e,g,i)** 250 μm; **(b,d,f,h,j)** 25 μm. **(B)** Quantitative analysis of optical density of Cx43 immunopositive cells in CA1 (*n* = 6 in each group). **(C)** Western blot analysis of Cx43 in CA1 of tGCI and HPC rats. The histogram presents the quantitative analyses of Cx43 levels (*n* = 3 in each group). Data are expressed as percentage of value of Sham animals. Each bar represents the mean ± SD *p < 0.05 vs. Sham animals and ^#^*p* < 0.05 vs. tGCI group at the same time point. Sham: sham-operated; tGCI: transient global cerebral ischemia; HPC: hypoxic preconditioning; Cx43: connexin 43.

Immunohistochemistry showed that the expression of Cx43 in CA1 was found in cell bodies and processes (Figures 5Aa–j). Compared with Sham rats, the intensities of Cx43 immunoreactivity in CA1 were significantly reduced at 4 and 24 h and enhanced at 168 h of reperfusion after tGCI. In contrast, there was no significant difference in Cx43 immunoreactivity between HPC and Sham rats (Figure 5B). In parallel with above-mentioned findings, similar results were observed in the level of Cx43 protein in CA1 after tGCI. But when rats were pretreated with hypoxia, the decrease in the Cx43 protein induced by tGCI was prevented at 4–48 h after reperfusion (Figure 5C).

**Figure 6:**
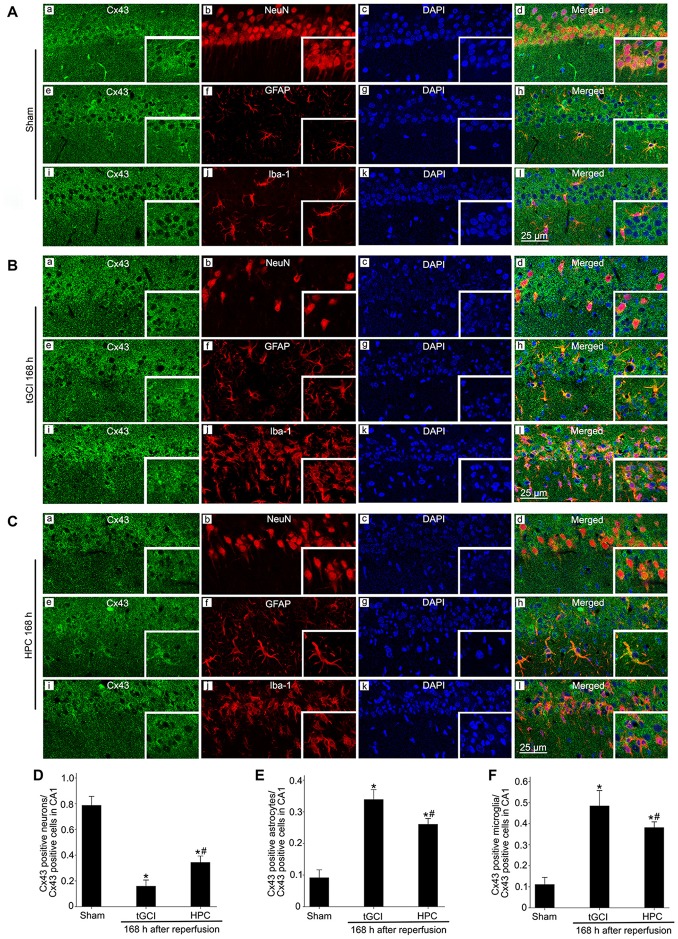
Cellular localization of Cx43 in CA1 in Sham group and tGCI group with or without hypoxia at 168 h of reperfusion. Representative images of triple fluorescent staining of Cx43 (green), NeuN (red) and DAPI (blue); Cx43 (green), GFAP (red) and DAPI (blue); Cx43 (green), Iba-1 (red) and DAPI (blue) in CA1. **(A)** The overlapped images showed that Cx43 prominently surrounded NeuN **(a–d)**, and located slightly in GFAP-positive **(e–h)** and Iba-1-positive **(i–l)** cells in Sham group. **(B)** Cx43 mainly located in GFAP-positive **(e–h)** and Iba-1-positive **(i–l)** cells at 168 h after tGCI, and part of cells with NeuN **(a–d)**. **(C)** Cx43 located mainly in GFAP-positive **(e–h)** and Iba-1-positive **(i–l)**, and partly in NeuN-positive cells **(a–d)** in CA1 at 168 h after reperfusion of HPC group. Scale bar: 25 μm. **(D–F)** Quantitative analysis of Cx43 positive neurons, Cx43 positive astrocytes and Cx43 positive microglia in the CA1, respectively. Data are expressed as percentage of the number of Cx43-positive cells. Each bar represents the mean ± SD; *p < 0.05 vs. Sham group and ^#^*p* < 0.05 vs. tGCI group (*n* = 4 in each group). Sham, sham-operated; tGCI, transient global cerebral ischemia; HPC, hypoxic preconditioning; Cx43, connexin 43; NeuN, neuronal nuclei; GFAP, glial fibrillary acidic protein; Iba-1, ionized calcium binding adaptor molecule 1; DAPI, 4’,6-diamidino-2-phenylindole.

In addition, the results with triple-fluorescent immunohistochemistry showed that in Sham group the cells surrounded by Cx43 positive labellings were primarily NeuN-positive (Figures 6Aa–d). Meanwhile, a minority of Cx43 positive cells colocalized with GFAP (Figures 6Ae–h) and Iba-1 (Figures 6Ai–l). Notably, at 168 h after reperfusion of tGCI, Cx43 positive cells mainly colocalized with Iba-1 (Figures 6Bi–l) and GFAP (Figures 6Be–h), a minority of Cx43 positive cells colocalized with NeuN (Figures 6Ba–d), indicating that most of cells are glia in tGCI rats. Alternatively, most of Cx43 positive cells co-localized with GFAP (Figures 6Ce–h) and Iba-1 (Figures 6Ci–l), and part of cells with NeuN and none of them with GFAP (Figures 8Ce–h) in HPC group (Figures 6Ca–d). The quantitative analysis of NeuN-, GFAP- and Iba-1 positive cells were shown in Figures 6D–F, respectively. Additionally, we conducted triple fluorescent staining of NeuN, Iba-1 and Cx43 in CA1. Cx43-positive cells were mainly colocalized with NeuN and a few with Iba-1 in sham-operated group (Supplementary Figures S1a–d). Cx43-positive cells were mainly colocalized with Iba-1, and a few with NeuN at 168 h after reperfusion (Supplementary Figures S1e–h). However, Cx43-positive cells were colocalized with both NeuN and Iba-1 at 168 h after reperfusion of HPC group (Supplementary Figures S1i–l).

**Figure 7:**
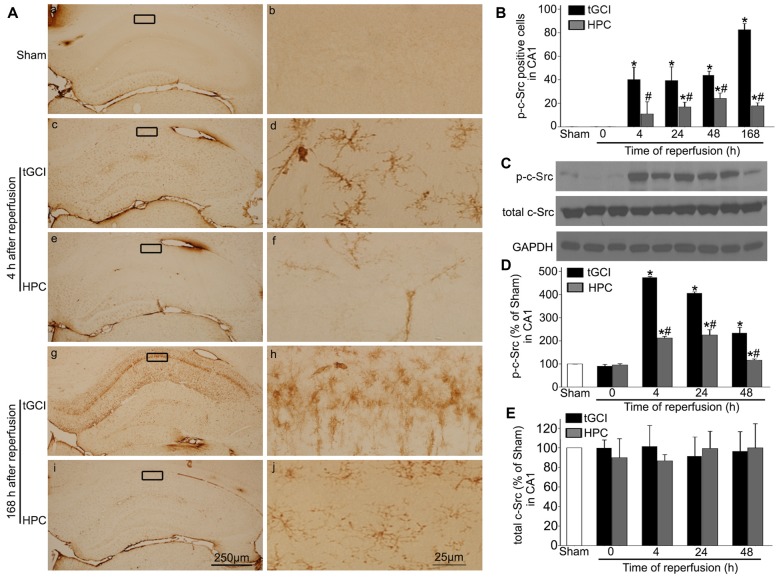
HPC decreases p-c-Src expression in CA1 after tGCI. **(A)** Immunohistochemistry for p-c-Src in hippocampus after tGCI with or without HPC. Representative images show p-c-Src immunopositive cells in Sham group **(a,b)**, 4 h after reperfusion of tGCI group **(c,d)** and HPC group **(e,f)**, and 168 h after reperfusion of tGCI group **(g,h)** and HPC group **(i,j)**, respectively. Scale bar: **(a,c,e,g,i)** 250 μm; **(b,d,f,h,j)** 25 μm. **(B)** Quantitative analysis of immunoreactive cell of p-c-Src in CA1 (*n* = 6 in each group). **(C)** Representative images of western blot of c-Src and p-c-Src in CA1 of tGCI and HPC rats. Quantitative analyses of p-c-Src protein **(D)** and total c-Src protein **(E)** in CA1. Data are expressed as percentage of value of Sham animals (*n* = 3 in each group). Each bar represents the mean ± S.D. *p < 0.05 vs. Sham animals and ^#^*p* < 0.05 vs. tGCI group at the same time point. Sham, sham-operated; tGCI, transient global cerebral ischemia; HPC, hypoxic preconditioning; c-Src, cellular-Src; p-c-Src, phosphorylated cellular-Src.

**Figure 8:**
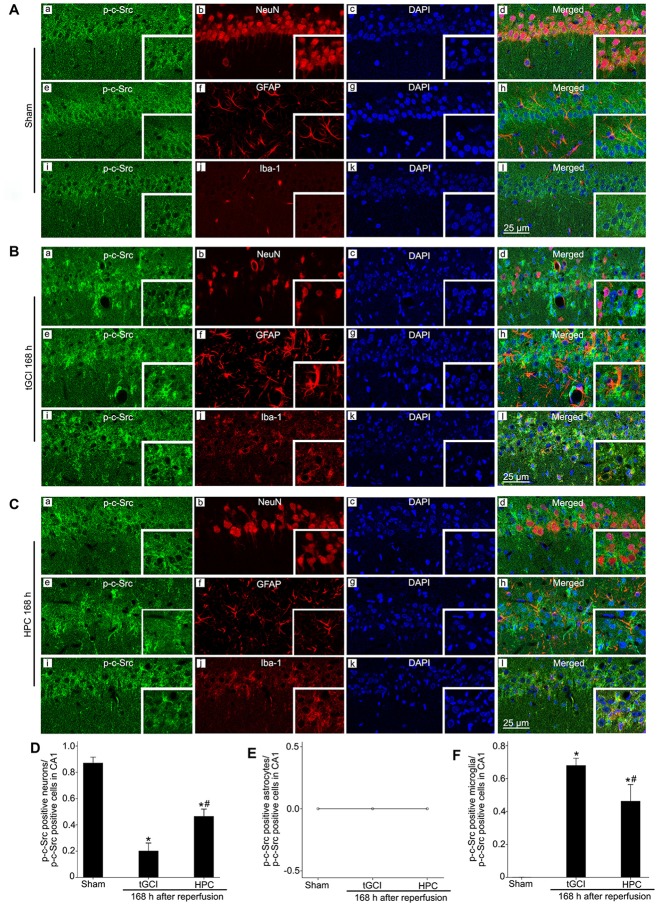
Cellular localization of p-c-Src in CA1 in Sham group and tGCI group with or without hypoxia at 168 h of reperfusion. Representative images of triple fluorescent staining of p-c-Src (green), NeuN (red) and DAPI (blue); p-c-Src (green), GFAP (red) and DAPI (blue); p-c-Src (green), Iba-1 (red) and DAPI (blue) in CA1. **(A)** The overlapped images showed that p-c-Src was mainly colocalized with NeuN positive cells **(a–d)**, rather than GFAP **(e–h)** and Iba-1 **(i–l)** positive cells in Sham group. **(B)** p-c-Src mainly located in Iba-1-positive **(i–l)** cells at 168 h after tGCI, and a minority of p-c-Src positive cells were NeuN-positive **(a–d)**, rather than GFAP-positive **(e–h)**. **(C)** p-c-Src located mainly in NeuN-positive **(a–d)** and Iba-1 positive **(i–l)** cells, but not in GFAP-positive **(e–h)** cells in CA1 at 168 h after reperfusion of HPC group. Scale bar: 25 μm. **(D–F)** Quantitative analysis of p-c-Src positive neurons, p-c-Src positive astrocytes and p-c-Src positive microglia in CA1, respectively (*n* = 4 in each group). Data are expressed as percentage of the number of p-c-Src-positive cells. Each bar represents the mean ± SD *p < 0.05 vs. Sham group and ^#^*p* < 0.05 vs. tGCI group. Sham, sham-operated; tGCI, transient global cerebral ischemia; HPC, hypoxic preconditioning; p-c-Src, phosphorylated cellular-Src; NeuN, neuronal nuclei; GFAP, glial fibrillary acidic protein; Iba-1, ionized calcium binding adaptor molecule 1; DAPI, 4’,6-diamidino-2-phenylindole.

The phosphorylation of c-Src at Tyr-416 (p-c-Src) was measured to analyze c-Src activity. Faint p-c-Src staining in CA1 from Sham animals was revealed by immunohistochemistry (Figures 7Aa–b). During 4–168 h after reperfusion of tGCI with or without hypoxia, p-c-Src-positive cells displayed an irregular appearance with polymorphic somata and processes, exhibiting a typical glia-like morphology in CA1 (Figures 7Ac–j). Meanwhile, the number of p-c-Src-positive cells was much higher in tGCI groups than in Sham group, while it was substantially decreased after HPC (Figure 7B). This changed trend of p-c-Src level in CA1 were further confirmed with western blot, while the total amount of c-Src protein remained constant (Figures 7C–E).

Triple-label immunofluorescence studies revealed that p-c-Src-positive cells colocalized with NeuN (Figures 8Aa–d), but not with GFAP (Figures 8Ae–h) or Iba-1 (Figures 8Ai–l), suggesting that p-c-Src predominantly localized in neurons in Sham group. Notably, at 168 h after reperfusion of tGCI, p-c-Src positive cells mainly colocalized with Iba-1 (Figures 8Bi–l), and a minority of them were NeuN-positive (Figures 8Ba–d), rather than GFAP-positive (Figures 8Be–h), indicating that these cells are microglia in tGCI rats. In addition, most of p-c-Src positive cells colocalized with Iba-1 (Figures 8Ci–l), and part of them with NeuN and none of them with GFAP (Figures 8Ce–h) in HPC group (Figures 8Ca–d). The quantitative analysis of NeuN-, GFAP- and Iba-1 positive cells were shown in Figures 8D–F, respectively.

**Figure 9:**
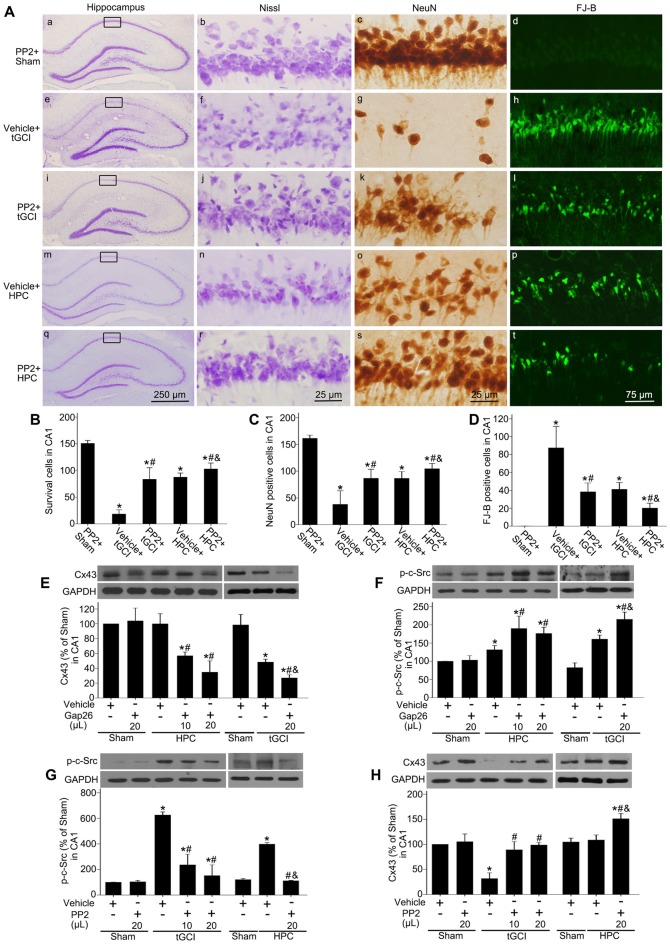
Inhibition of c-Src activity with PP2 alleviates neuronal damage in CA1 of tGCI rats with or without hypoxia. **(A)** Nissl staining, NeuN immunostaining and FJ-B staining from tGCI and HPC rats with or without PP2 treatment at 168 h after reperfusion. Boxes indicated that the magnified regions displayed in the right panel. Scale bar: **(a,e,i,m,q)**: 250 μm; **(b,c,f,g,j,k,n,o,r,s)** 25 μm; **(d,h,l,p,t)** 75 μm. Quantitative analyses of survival cells **(B)**, NeuN-positive cells **(C)**, and FJ-B positive cells **(D)** in CA1. Each bar represents the mean ± SD *p < 0.05 vs. Sham group pretreated with PP2, ^#^*p* < 0.05 vs. the same group with vehicle, and ^&^*p* < 0.05 vs. tGCI group with PP2 injection (*n* = 6 in each group). The effect of Gap26 (10 μL and 20 μL, respectively) on Cx43 expression **(E)** and p-c-Src level **(F)** in CA1 at 4 h after reperfusion in tGCI and HPC group. The effect of PP2 (10 μL and 20 μL, respectively) on p-c-Src level **(G)** and Cx43 expression **(H)** in CA1 at 4 h after reperfusion in tGCI and HPC group. Data are expressed as the percentage of value of Sham group with vehicle. Each bar represents the mean ± SD *p < 0.05 vs. Sham group with vehicle, ^#^*p* < 0.05 vs. the same group with vehicle, and ^&^*p* < 0.05 vs. HPC group with Gap26 (*n* = 3 in each group; **E,F**) or tGCI group with PP2 (*n* = 3 in each group; **G,H**). Sham, sham-operated; tGCI, transient global cerebral ischemia; HPC, hypoxic preconditioning; p-c-Src, phosphorylated cellular-Src; Cx43, connexin 43; Gap26, Cx43 mimetic peptide, PP2, 4-amino-5-(4-chlorophenyl)-7-(t-butyl) pyrazolo (3, 4-d) pyrimidine; Nissl, cresyl violet; NeuN, neuronal nuclei; FJ-B: Fluoro-Jade B.

To evaluate the effects of c-Src on the neuronal death after tGCI with or without hypoxia, PP2 was administered before tGCI or HPC. As shown in Figure 9A, PP2 had no any neurotoxic effects on the cells of hippocampus in Sham group (Figures 9Aa–d). Compared with the vehicle groups (Figures 9Ae–h,m–p), the number of survival cell and NeuN-positive cell significantly increased, whereas FJ-B-positive cell markedly decreased in the rats with PP2 pretreatment either in tGCI or HPC groups, but the neuroprotective effects from HPC groups were stronger (Figures 9Ai–l,q-t,B–D).

To study the association between c-Src and Cx43 after tGCI with or without hypoxia, either Gap26 or PP2 was administered before tGCI or HPC. In comparison to the vehicle groups the blockage of Cx43 with Gap26 (Figure 9E) resulted in an increase of p-c-Src in CA1 at 4 h after reperfusion in the rats either in tGCI or HPC groups, but the former was much more obvious (Figure 9F), whereas the inactivation of c-Src with PP2 (Figure 9G) enhanced Cx43 protein expression in CA1 in tGCI and HPC rats, with the latter changing more noticeable (Figure 9H).

### Hypoxic Preconditioning Maintains GLT-1 Expression in CA1 Through Upregulation Of Cx43 and Inhibition of c-Src After tGCI

**Figure 10:**
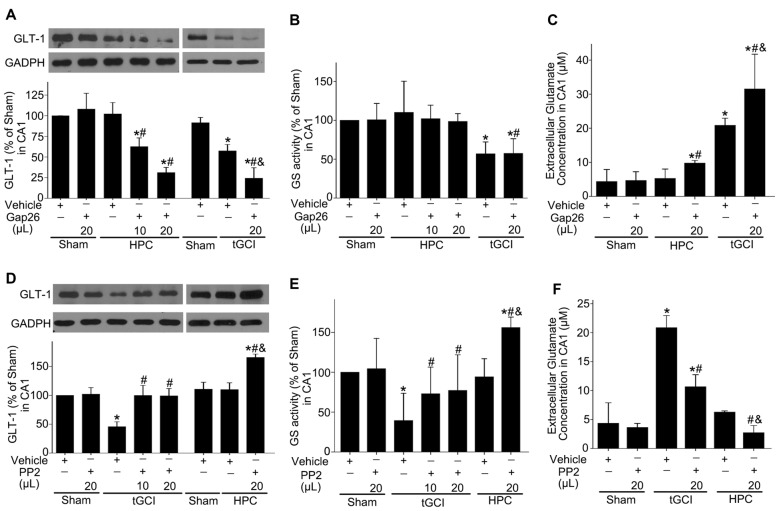
Effect of Gap26 or PP2 on the expression of GLT-1 and GS activity in CA1 of rats exposed to tGCI or HPC. The effect of Gap26 on GTL-1 expression **(A)** and GS activity **(B)** in CA1 at 4 h after reperfusion in tGCI and HPC group. **(C)** The effect of Gap26 on extracellular glutamate in CA1 at 24 h after reperfusion in tGCI and HPC group. The effect of PP2 on GLT-1 expression **(D)** and GS activity **(E)** in CA1 at 4 h after reperfusion in tGCI and HPC rats. **(F)** The effect of PP2 on extracellular glutamate at 24 h after reperfusion in tGCI and HPC rats. Each bar represents the mean ± S.D. *p < 0.05 vs. Sham group, ^#^*p* < 0.05 vs. the same group with vehicle, and ^&^*p* < 0.05 vs. HPC group with Gap26 **(A–C)** or tGCI group with PP2 (**D–F**; *n* = 3 in each group). Sham, sham-operated; tGCI, transient global cerebral ischemia; HPC, hypoxic preconditioning. GS, glutamine synthetase; GLT-1, glutamate transporter 1; Gap26, Cx43 mimetic peptide, PP2, 4-amino-5-(4-chlorophenyl)-7-(t-butyl) pyrazolo (3, 4-d) pyrimidine.

Afterwards, we explored the effects of Cx43 and c-Src on the expression of GLT-1, GS activity and the concentration of extracellular glutamate in CA1 after tGCI with or without hypoxia. As shown in Figure 10, compared with the vehicle groups, Gap26 decreased GLT-1 expression and exerted no any effect on GS activity (Figures 10A,B), but increased the level of extracellular glutamate in CA1, especially in the group of tGCI alone (Figure 10C). In addition, PP2 led to a remarkable increase in GLT-1 expression and GS activity in CA1 at 4 h, and it lead to a significant decrease in extracellular glutamate level at 24 h after tGCI. These trends in HPC were more apparent than in tGCI group (Figures 10D–F). These findings indicate that HPC maintained GLT-1 expression by up-regulating Cx43 expression, whereas the decrease of p-c-Src level substantially blocked the descended expression of GLT-1 and GS activity after tGCI.

## Discussion

**Figure 11:**
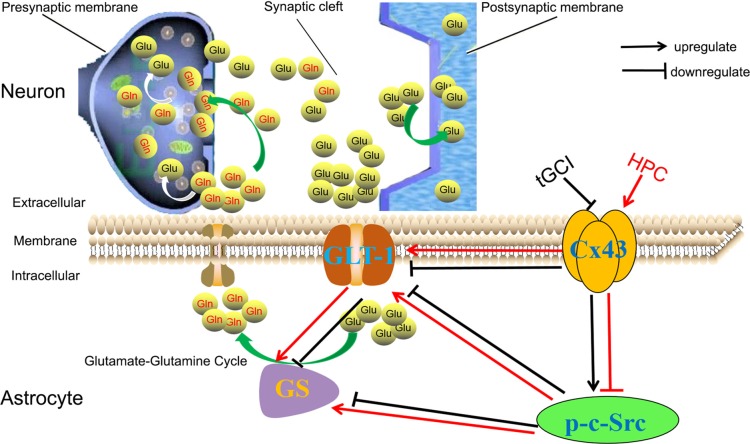
HPC regulates GLT-1 and the activity of GS against tGCI through regulation of Cx43 and c-Src. Generally, glutamates (Glu) can be synthesized inside the presynaptic terminal and then released into the synaptic cleft. Most Glu are transported into astrocytes by GLT-1 and shall be converted to glutamine (Gln) by GS and released into the extracellular space. Subsequently, Gln shall be uptaken into the presynaptic terminals and metabolized to Glu, the process of which is termed glutamate-glutamine cycle. Under physiological conditions, extracellular glutamate concentration shall be maintained at a proper level. However, under ischemic conditions, the decreased expression of Cx43 and /or the increased c-Src activity can decrease the expression of GLT-1 and reduce the activity of GS, and then disrupt the operation of glutamate-glutamine cycle, leading to the accumulation of extracellular glutamate. HPC can prevent the reduction of GLT-1 expression and maintain GS activity by regulating Cx43 and inactivating c-Src to remove the released glutamate from the extracellular space, eventually protecting neurons against tGCI. HPC, hypoxic preconditioning; tGCI, transient global cerebral ischemia; Glu, glutamate; Gln, glutamine; Cx43, connexin 43; c-Src, cellular-Src; p-c-Src, phosphorylated cellular-Src; GLT-1, glutamate transporter 1; GS, glutamine synthetase.

As shown in Figure 11, our study supported the hypothesis that HPC prevents the increase in extracellular glutamate and protects against tGCI-induced neuronal death in hippocampal CA1 by maintaining of GLT-1 and GS activity. These effects following HPC may result from an increase of Cx43 expression and a decrease in c-Src activity.

As a key factor in the cascade leading to cerebral ischemic injury, glutamate excitotoxicity is considered as the triggering spark. In the present study, *in vivo* microdialysis revealed significantly increase in extracellular glutamate in CA1 after tGCI, which is consistent with previous findings (Liu et al., 2012; Gong et al., 2014; Guo et al., 2015). It is a well-established fact that the extracellular glutamate homeostasis in the central nervous system (CNS) is mainly regulated by GLT-1 (Kim et al., 2011). Also, impaired glutamate uptake into astrocytes induced by dysfunction or reduced expression of GLT-1 has been implicated in the pathogenesis of cerebral ischemia (Ketheeswaranathan et al., 2011; Fang et al., 2014). Additionally, downregulation of GLT-1 followed by accumulation of glutamate in extracellular fluid (Lehmann et al., 2009) has been confirmed in several cerebral ischemic models both *in vivo* and *in vitro* (Rao et al., 2001; Chen et al., 2005; Vandresen-Filho et al., 2013; Gong et al., 2014; Dal-Cim et al., 2016). Consistent with the observations of other studies (Rao et al., 2001; Chen et al., 2005; Gong et al., 2014), our study demonstrated increased extracellular glutamate and decreased GLT-1 protein in CA1 after tGCI, whereas these trends were reversed when pre-treated with hypoxia. Moreover, when blocking GLT-1 with DHK, the accumulated extracellular glutamate after ischemia was aggravated, and the neuroprotection mediated by HPC was prevented. These results were similar to previous reports (Fang et al., 2014; Sun et al., 2015). Hence, we speculate that HPC increased the uptake of extracellular glutamate into astrocytes and protected neurons against tGCI by maintaining GLT-1 expression in CA1 after tGCI.

GLT-1 can be substantially expressed in the hippocampus and cortex and involved in the extracellular glutamate clearance in the adult forebrain (Holmseth et al., 2012). In the current study, the immunoreactivity of GLT-1 in HPC groups at 4–24 h and 168 h of reperfusions were much higher than that in tGCI groups, and most GLT-1 positive cells were predominantly astrocytes. This pattern of GLT-1 distribution was proven to be consistent with previous study (Liu et al., 2012). Also, astrocytes can dynamically regulate synaptic transmission by expressing varieties of neurotransmitter receptors and releasing several types of neurotransmitters, including glutamate (Newman, 2003). In this study, we found that most reactive astrocytes were characterized by hypertrophic cell bodies and thick processes in tGCI groups, whereas in HPC groups these astrocytes exhibited quiescent phenotype with a stellate, process-bearing shape. Reactive astrogliosis that performed as enlarged cell bodies and branches brought about a decrease in GLT-1 expression in the hippocampus of a mouse model with Alzheimer’s disease that was established by ovariectomy (Liu et al., 2010). Also, previous studies have demonstrated that quiescent astrocytes express higher level of GLT-1 (Tawfik et al., 2006). Hence, the preventing reduction of GLT-1 induced by HPC after tGCI may be mainly attributed to astrocytes kept in a quiescent phenotype.

Regulated coordination between glutamate uptake and glutamate degradation was recognized to be essential for effective glutamate-glutamine cycle in glial cells (Rauen and Wiessner, 2000). When exposed to global brain ischemia, the expression of GS in the hippocampus of rats decreased, but increased significantly with ischemic postconditioning (Zhang et al., 2011). However, our results failed to demonstrate any effect of HPC or tGCI on the expression of GS during reperfusion. Notably, GS activity was found to be distinctly higher in HPC rats than in tGCI groups, and the inhibition of GS activity by MSO before HPC induced an accumulation of extracellular glutamate. These results implied that HPC can buffer the excess glutamate by maintaining GS activity after tGCI, thereby mediating neuronal survival. In our study, the inhibition of GS activity with MSO exerted no effect on GLT-1 expression, whereas the inhibition of GLT-1 with DHK could decrease GS activity induced by HPC. A recent study from Das showed that the increased GS activity in nucleus accumbens (NAc) of male alcohol-preferring rats with ceftriaxone treatment can be attributed to the maintenance of glutamate inflow by increasing GLT-1 expression within astrocytes (Das et al., 2015). Trabelsi et al. (2017) has demonstrated that the inactivation of GS resulted in cytosolic accumulation of glutamate, hinting at the critical role in the conversion of glutamate by GS for excitatory amino acid transporters (EAATs) operation. Even though our study exhibited no effect on the expression of GLT-1 with MSO, the fact that GS affects GLT-1 efficiency rather than its expression may explain this phenomenon.

It has been proposed that GLT-1 level depends on the expression of GJs proteins (Figiel et al., 2007). As a major astrocytic GJ protein in astrocytes, Cx43 acts as the most abundant connexins in mammals. The data from Nakase et al. (2006) suggested that neuroprotection was induced in human brain by increasing the levels of Cx43 after ischemic stroke. Pannasch et al. (2011) reported that astrocytes in Cx30^−/–^ Cx43^−/–^ mice failed to properly remove enhanced extracellular glutamate, leading to prolong neuronal excitatory activity. Our study showed that the expression of Cx43 in CA1 was decreased at 4–24 h after cerebral ischemia, consistent with other studies (Li et al., 2005; Haupt et al., 2007), whereas the immunoreactivity of Cx43 in CA1 notably increased at 168 h after tGCI. It was reported that ischemia-induced acidification of astrocytes reduces intercellular communication by closing GJ channels and subsequently internalizing GJ proteins (Morley et al., 1996; Yokota et al., 2000; Skatchkov et al., 2015). The evidences from Duffy et al. (2004) showed that intracellular acidification of astrocytes led to internalization of Cx43 protein, with an enhanced association of Cx43 to c-Src and a loss of association of Cx43 to Zonula Occludens-1. Our results implied that the downregulation of Cx43 expression at early stage after tGCI may be attributed to the internalization of Cx43 and its upregulation at later stage to the enhancement of astrocytic gap junctional intercellular communication. In the CNS, astrocytes establish a glial syncytium through GJs (Kielian, 2008). In this process, blockade of Cx43 can compromise the astrocytic syncytium and impair vital functions including glutamate uptake from the extracellular space via the transporters i.e., GLT-1 and glutamate aspartate transporter (GLAST), and conversion of glutamate to glutamine as well (Davidson et al., 2012). Accordingly, the inhibition of Cx43 by Gap26 can directly affect astrocytic syncytium and downregulate GLT-1, thus leading to accumulation of extracellular glutamate. It has been noted that the current study fails to account for why the inhibition of Cx43 exerted no effect on the GS activity at the same time. Hence, further studies are needed to study the relation between Cx43 and GS activity.

Accumulated evidences showed that an important function of Src is to regulate the activity of *N*-methyl-D-aspartic acid (NMDA) receptors and other ion channels. Src is located in post-synaptic NMDA receptor complex (Zhang F. et al., 2007). The increased Src kinase activity in the paraventricular nucleus of the hypothalamus plays a pivotal role in increasing pre- and postsynaptic NMDA receptor activity of presympathetic neurons in spontaneously hypertensive rats, which may facilitate glutamate release (Qiao et al., 2017). In this study, the p-c-Src level performed a significant increase at 4–48 h after tGCI, but this process was reversed by HPC. Further, the inhibition of c-Src with PP2 reduced extracellular glutamate level and exerted a significant protective effect on CA1 neurons after tGCI. Taken together, HPC prevented the accumulation of extracellular glutamate and attenuated neuronal death against tGCI through inhibiting c-Src kinase activity.

The association between c-Src and Cx43 has not been fully elucidated. So far there has been no study examining whether this link takes place in reactive astrocytes after cerebral ischemic injury. Cx43 and Src are mutually regulated by a phosphorylation/dephosphorylation loop. As an interacting partner of Cx43 COOH-terminal tail, the activated c-Src phosphorylates Cx43 on critical residues Tyr247 and Tyr265 which inhibits gap junctional intercellular communication (Giepmans et al., 2001; Herrero-González et al., 2010; Tabernero et al., 2016). In reverse, as a substrate of c-Src, the upregulation of Cx43 in glioma cells reduced the c-Src activity by decreasing phosphorylated c-Src at tyrosine 416 and by increasing the inactive form of Src (phosphor-Src Tyr527; Herrero-González et al., 2010; Gangoso et al., 2014), while downregulation or silencing of Cx43 activated c-Src promoted proliferation and increased the rate of glucose uptake (Gangoso et al., 2012; Valle-Casuso et al., 2012).

Similarly, we observed a lower expression of Cx43 and a higher level of p-c-Src in CA1 after cerebral ischemia. The inhibition of c-Src with PP2 increased Cx43 in ischemic rats. Interestingly, HPC maintained the expression of Cx43 and downregulated the level of p-c-Src after tGCI, which was similar to that promoted by PP2. These data suggest that HPC by inhibiting c-Src activity protected against cerebral ischemia. The inhibition of Cx43 with Gap26 dramatically increased p-c-Src level in the rats after tGCI, which may inhibit gap junctional intercellular communication and in turns abrogates the neuroprotective effects of HPC. Previously, Hou et al. (2007) demonstrated that PP2 markedly increased the number of surviving pyramidal neurons in CA1 of rats after tGCI. Having confirmed the foregoing hypothesis, the current study also hints at the interaction between Cx43 and c-Src being an important step toward the neuroprotection induced by HPC after tGCI. Also, the c-Src activation coincided with the reduction in Cx43 expression. For the first time we demonstrated that HPC can increase GLT-1 expression and maintain GS activity by up-regulating Cx43 and inactivating c-Src to remove the released glutamate from the extracellular space in the ischemic brain.

Notably, the four inhibitors (including DHK, MSO, Gap26 and PP2) used in our study had no impact on Sham rats, compared to the inhibited effects on HPC or tGCI rats. There might be three reasons. First, under physiological state, Sham rats are able to resist the effects of those inhibitors, properly through self-compensation. Second, unlike physiological conditions those inhibitors may play their roles through regulating different signaling pathways, either activated or inactivated after tGCI with or without hypoxia. Third, some inhibitors inhibit the activity of substrates that are only stimulated but not autonomous. It is still unclear whether this observation is linked to the mechanisms described above and it warrants further investigation.

In summary, we have showed that HPC prevented the accumulation of extracellular glutamate and attenuated neuronal death against tGCI in adult Wistar rats by maintaining GLT-1 expression and GS activity in hippocampal CA1, which is attributable to the upregulation of Cx43 expression and inhibition of c-Src activity. Our new findings indicate that HPC seems to be able to offer a potential strategy through modulation of GLT-1 in order to prevent glutamate excitotoxicity and to exert neuroprotection to cerebral ischemia.

## Author Contributions

EX and LZ designed the experiments. KL, HZ and LZ collected the data. KL, HZ, LL, DDL, ZS, YT and WX prepared the samples from four-vessel tGCI-reperfusion rat model, and performed all the experiments related to histology, immunohistochemistry and western blot. KL, ZS, YT and WX contributed to microdialysis and drug injection. KL and ZS performed assay of GS activity. WS performed the experiments related to the double-fluorescent staining of immunohistochemistry. The manuscript was prepared by EX and KL with assistance from LZ and DHL. All authors read and commented on the manuscript and approved the final version of the manuscript.

## Conflict of Interest Statement

The authors declare that the research was conducted in the absence of any commercial or financial relationships that could be construed as a potential conflict of interest.

## Abbreviations

BCA: bicinchoninic acid
CA1: Cornu Ammonis 1
CNS: central nervous system
c-Src: cellular-Src
Cx43: Connexin 43
DHK: dihydrokainate
DMSO: Dimethylsulfoxide
EAAT: Excitatory amino acid transporter
FJ-B: Fluoro-Jade B
Gap26: Cx43 mimetic peptide
GFAP: Glial fibrillary acidic protein
GJ: gap junction
GLAST: Glutamate aspartate transporter
GLT-1: Glutamate transporter 1
GS: Glutamine synthetase
HPC: Hypoxic preconditioning
HPLC: High-performance liquid chromatography
I/R: ischemia-reperfusion
Iba-1: Ionized calcium binding adaptor molecule-1
MSO: Methionine sulfoximine
NeuN: Neuronal nuclei
Nissl: Cresyl violet
NMDA: *N*-methyl-D-aspartic acid
PP2: 4-amino-5-(4-chlorophenyl)-7-(t-butyl) pyrazolo (3,4-d) pyrimidine
PVDF: polyvinylidene fluoride
SD: Standard Deviation
sham: sham-operated
tGCI: Transient global cerebral ischemia.
