# Supplementary material for: Hypoxic Preconditioning Maintains GLT-1 Against Transient Global Cerebral Ischemia Through Upregulating Cx43 and Inhibiting c-Src
Source: Front Mol Neurosci. 2018 Oct 1;11:344. doi: 10.3389/fnmol.2018.00344 (PMC6172853; doi:10.3389/fnmol.2018.00344)
Supplement: Supplementary file 1 [file Data_Sheet_1.doc]

Supplementary Material

Hypoxic Preconditioning Maintains GLT-1 against Transient Global Cerebral Ischemia through Upregulating Cx43 and Inhibiting c-Src

**Kongping Li, Huarong Zhou, Lixuan Zhan, Zhe Shi, Weiwen Sun, Dandan Liu, Liu Liu, Donghai Liang, Yafu Tan, Wensheng Xu, En Xu***

*** Correspondence:** Dr. En Xu: [enxu@163.net](mailto:enxu@163.net.com)

# Supplementary Figures and Tables

## Supplementary Figures


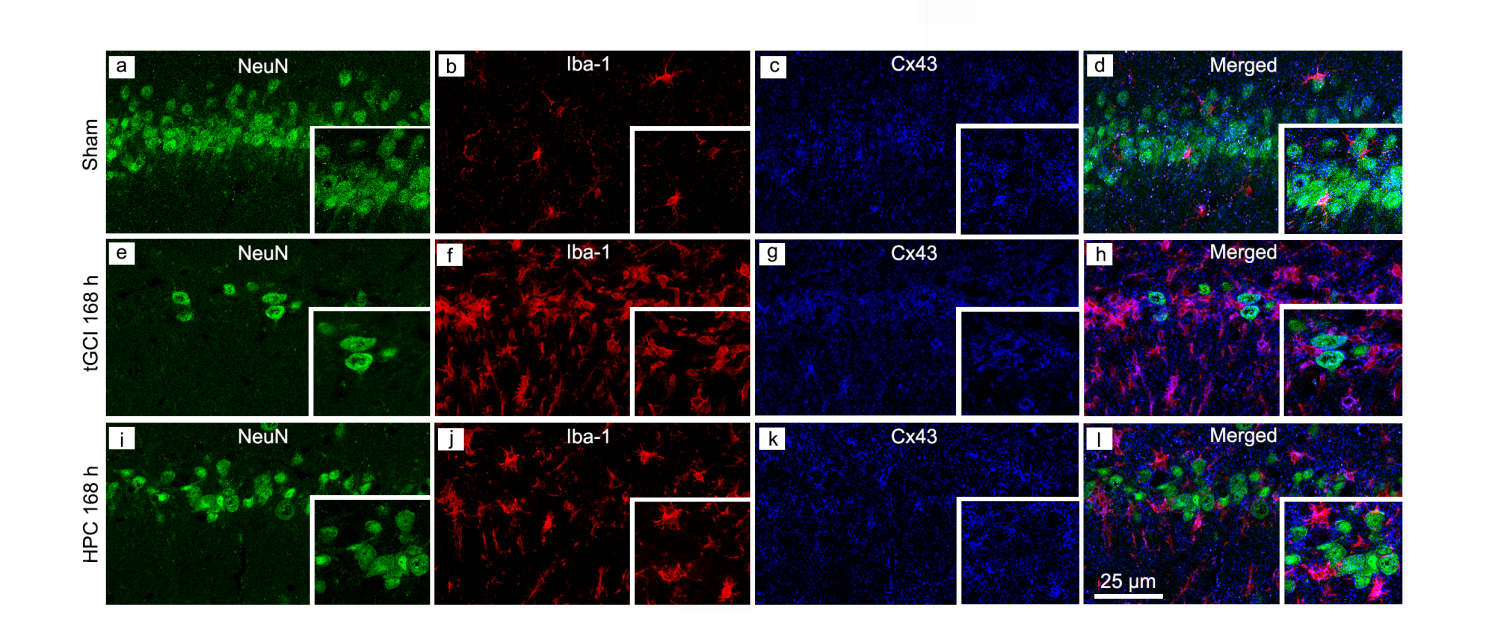


**Supplementary Figure 1.** Cellular localization of Cx43 in CA1 in sham-operated group and tGCI group with or without hypoxia at 168 hours of reperfusion. Representative images of triple fluorescent staining of NeuN (green), Iba-1 (red) and Cx43 (blue) in CA1. Cx43-positive cells (blue) were mainly colocalized with NeuN (green) and a few with Iba-1 (red) in sham-operated group (a-d). Cx43-positive cells (blue) were mainly colocalized with Iba-1 (red), and a few with NeuN (green) (e-h) at 168 hours after reperfusion. However, Cx43-positive cells (blue) were colocalized with both NeuN (green) and Iba-1(red) at 168 hours after reperfusion of HPC group (i-l). Scale bar: 25 μm.
